# Self-Assembled Supramolecular Nanoparticles Improve the Cytotoxic Efficacy of CK2 Inhibitor THN7

**DOI:** 10.3390/ph11010010

**Published:** 2018-01-26

**Authors:** Abdelhamid Nacereddine, Andre Bollacke, Eszter Róka, Christelle Marminon, Zouhair Bouaziz, Ferenc Fenyvesi, Ildikó Katalin Bácskay, Joachim Jose, Florent Perret, Marc Le Borgne

**Affiliations:** 1 Faculté de Pharmacie—ISPB, EA 4446 Bioactive Molecules and Medicinal Chemistry, SFR Santé Lyon-Est CNRS UMS3453—INSERM US7, Université de Lyon, Université Claude Bernard Lyon 1, 8 Avenue Rockefeller, F-69373 Lyon CEDEX 8, France; a.nacereddine@gmail.com (A.N.); christelle.marminon-davoust@univ-lyon1.fr (C.M.); zouhair.bouaziz@univ-lyon1.fr (Z.B.); 2 Institute of Pharmaceutical and Medicinal Chemistry, PharmaCampus, Westfälische Wilhelms-Universität Münster, Corrensstr. 48, 48149 Münster, Germany; andre.bollacke@uni-muenster.de (A.B.); joachim.jose@uni-muenster.de (J.J.); 3 Department of Pharmaceutical Technology, Faculty of Pharmacy, University of Debrecen, Nagyerdei körút 98, H-4032 Debrecen, Hungary; eszter.roka@gmail.com (E.R.); fenyvesi.ferenc@pharm.unideb.hu (F.F.); bacskay.ildiko@pharm.unideb.hu (I.K.B.); 4 CSAp, Institut de Chimie et Biochimie Moléculaires et Supramoléculaires, Bâtiment Raulin, Université de Lyon, Université Lyon 1, 43 Bd du 11 novembre 1918, 69622 Villeurbanne CEDEX, France

**Keywords:** indeno[1,2-*b*]indole, CK2 inhibitor, cyclodextrin, nanoparticles, in cellulo, human erythrocytes, A427 cells

## Abstract

Since the approval of imatinib in 2001, kinase inhibitors have revolutionized cancer therapies. Inside this family of phosphotransferases, casein kinase 2 (CK2) is of great interest and numerous scaffolds have been investigated to design CK2 inhibitors. Recently, functionalized indeno[1,2-*b*]indoles have been revealed to have high potency against human cancer cell lines such as MCF-7 breast carcinoma and A-427 lung carcinoma. 4-Methoxy-5-isopropyl-5,6,7,8-tetrahydroindeno[1,2-*b*]indole-9,10-dione (THN7), identified as a potent inhibitor of CK2 (IC_50_ = 71 nM), was selected for an encapsulation study in order to evaluate its antiproliferative activity as THN7-loaded cyclodextrin nanoparticles. Four α-cyclodextrins (α-CDs) were selected to encapsulate THN7 and all experiments indicated that the nanoencapsulation of this CK2 inhibitor in α-CDs was successful. No additional surface-active agent was used during the nanoformulation process. Nanoparticles formed between THN7 and α-C_6_H_13_ amphiphilic derivative gave the best results in terms of encapsulation rate (% of associated drug = 35%), with a stability constant (K_11_) of 298 mol·L^−1^ and a size of 132 nm. Hemolytic activity of the four α-CDs was determined before the in cellulo evaluation and the α-C_6_H_13_ derivative gave the lowest value of hemolytic potency (HC_50_ = 1.93 mol·L^−1^). Only the THN7-loaded cyclodextrin nanoparticles showing less toxicity on human erythrocytes (α-C_6_H_13_, α-C_8_H_17_ and α-C_4_H_9_) were tested against A-427 cells. All drug-loaded nanoparticles caused more cytotoxicity against A-427 cells than THN7 alone. Based on these results, the use of amphiphilic CD nanoparticles could be considered as a drug delivery system for indeno[1,2-*b*]indoles, allowing an optimized bioavailability and offering perspectives for the in vivo development of CK2 inhibitors.

## 1. Introduction

The protein kinase CK2 is an ubiquitous and pleiotropic serine/threonine kinase with hundreds of endogenous substrates that are implicated in a wide variety of cellular functions (e.g., growth, proliferation, differentiation, apoptosis) [1]. CK2 is generally described as a tetrameric structure composed of two catalytic (α or α′) and two regulatory (β) subunits. CK2 is involved in several pathological processes [2] and it is especially linked to cancer such as leukemia [3,4], glioblastoma [5], and hepatocellular carcinoma [6].

**Figure 1:**
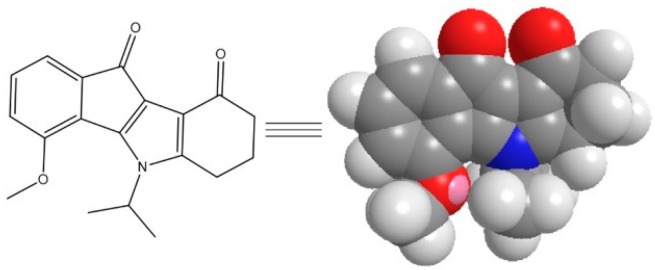
Structure of THN7 derivative.

Researchers are currently exploring a wide range of scaffolds to design small molecule inhibitors of CK2 [7]. Among these inhibitors, indeno[1,2-*b*]indole derivatives have been identified as ATP-competitive inhibitors of CK2 [8,9]. Structure-Activity Relationship (SAR) studies through the systematic modification of the four rings A-D brought to light the main positions that identified potent inhibitors of CK2. In fact, the introduction of an alkoxy group in position 4 (ring A) [10] or an alkyl group in position 7 [11] was associated with the most favorable inhibitory effects on CK2. The 4-methoxy-5-isopropyl-5,6,7,8-tetrahydroindeno[1,2-*b*]indole-9,10-dione (THN7, Figure 1) was identified as a potent inhibitor of CK2 (IC_50_ = 71 nM, unpublished data) and then selected for new encapsulation in a set of amphiphilic cyclodextrins (CDs). A “proof of concept” study was previously carried out and validated the use of these macrocyclic oligosaccharides as nanocarriers [12]. The investigation of drug formulations remains a key point in the use of bioactive molecules, especially for indenoindoles used as CK2 inhibitors.

Over the years, cyclodextrins became efficient vehicles to solubilize drugs and then to optimize diverse biological parameters (e.g., potency, absorption, distribution, metabolism, excretion, and toxicity (ADMET) properties) [13,14]. Moreover, amphiphilic cyclodextrin derivatives have been developed [15,16,17] in order to favor the interaction of CDs with cell membranes, to form self-assembled nanoparticles for drug encapsulation, enhancing interaction with hydrophobic drugs [18]. For example, amphiphilic cyclodextrins are useful to formulate stable nanoparticles of acyclovir, without the use of any surfactant [19].

**Figure 2:**
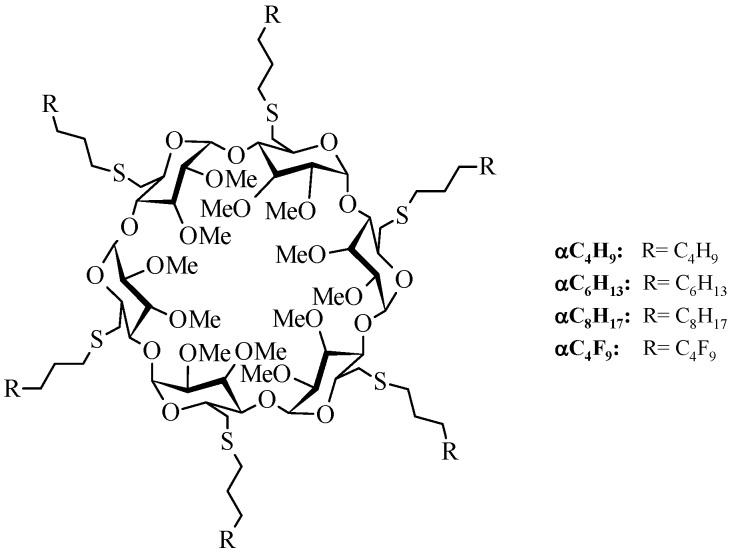
Fluorinated and hydrocarbonated amphiphilic α-cyclodextrins used in this study.

Hydrocarbonated and fluorinated amphiphilic α- and β-CDs derivatives were well studied by our group, and it has been shown that these molecules could form very stable nanoparticles that could entrap hydrophilic and hydrophobic drugs [20,21,22]. As demonstrated previously for an indeno[1,2-*b*]indole analog [12], nanoparticles made from these derivatives can not only encapsulate this CK2 inhibitor, but they can also release it in a controlled manner. We also demonstrated that nanoparticles made from α-CDs derivatives (Figure 2) are more resistant than those made from β-CDs derivatives, even if the complexations are weaker.

In this study, we investigated a new formulation study in which compound THN7 (cLogP 3.326, www.molinspiration.com/cgi-bin/properties, accession date: 16 September 2017) is directly loaded into α-cyclodextrin nanoparticles. Our works were for the first time completed by cell-based assays to demonstrate (i) the safety profile of the four α-cyclodextrins used as nanocarriers and (ii) the improved in cellulo activity of CK2 inhibitor THN7. Red blood cells (RBCs) and A-427 cell line were selected to achieve our goal.

## 2. Results

### 2.1. Characterization of THN7:Amphiphilic Cyclodextrin Inclusion Complexes

Due to its very simple utilization, UV/Vis spectroscopy is the method of choice for studying the complexation between amphiphilic α-cyclodextrin derivatives and UV-visible THN7 molecules in ethanol. It has also been chosen for comparison with previously obtained constants determined by the same technique [12]. By using Job and Benesi-Hildebrand plots, stoichiometry and stability constants (K_11_), respectively, can be easily and rapidly determined. All amphiphilic CD derivatives form 1:1 complexes with THN7 (maximum at 0.5 ratio) (Supplementary Information (SI) Figures S1–S4) and Benesi-Hildebrand plots are linear (SI Figure S5). The results obtained from these plots are given in Table 1.

The chain lengths and nature on the α-cyclodextrin derivatives seem to have an impact on the association at the molecular scale. Indeed, the K_11_ values increased with the chain length and were higher in the fluorinated derivative. For α-C_4_H_9_ the complexation was weak (57 mol·L^−1^), meaning that at the molecular level, it had no affinity for THN7. Nevertheless, by increasing the length of the hydrocarbonated chain, the affinity also increased (298 mol·L^−1^ and 417 mol·L^−1^ for α-C_6_H_13_ and α-C_8_H_17_, respectively), with the higher association constant being observed with the fluorinated analog α-C_4_F_9_ (828 mol·L^−1^). We also noticed that these values are in the same range as those observed previously by our group for another CK2 inhibitor. The differences in the behavior were quite similar: a higher association constant was observed with the fluorinated derivative [12]. All these observations also indicate that the THN7 molecule should be located between the hydrophobic chains rather than in the cyclodextrin cavity, as the molecule is too bulky to fit into the cavity.

### 2.2. THN7 Loading of Nanoparticles Based on Amphiphilic α-Cyclodextrins

The loading of THN7 compound into α-CDs nanoparticles was achieved as previously described [12,20]. Ethanol was used as an organic solvent because it has been shown that, among other solvents, for nanoparticle formation, ethanol gave the smallest particle size and better polydispersity [23]. At the end of the process, for each amphiphilic α-cyclodextrin used, a 20-mL stock solution of nanoparticles containing 600 μM THN7 was obtained and then stored before use.

Dynamic Light Scattering was employed for measuring sizes of the nanoparticles (SI Figures S6–S9) and the percentage of associated drug was determined after two days. The results are given in Table 2.

As seen in Table 2, the mean particle sizes varies from 66 to 132 nm. Unlike our previous observations of another CK2 inhibitor [12], for the same chain length, fluorinated nanoparticles are bigger (104 vs. 82 nm for α-C_4_F_9_ and α-C_4_H_9_, respectively). This may be due to the THN7 molecule′s better affinity for these fluorinated derivatives at the molecular scale, implying either more molecules inside the particles, or THN7 coating on the surface. Nevertheless, all of the particle size distributions are monodisperse, with a low polydispersity index (<0.2).

The loading capacities ranged from 14 to 35% (for α-C_8_H_17_ and α-C_6_H_13_, respectively). This is relatively low compared to the previous ones observed with another indenoindole derivative [12]. Nevertheless, it is worth noting that these encapsulation rates are still in the same range as those observed for other systems. No correlation between the loading capacities and the association constant determined at the molecular level was observed.

### 2.3. Controlled Release Studies

**Figure 3:**
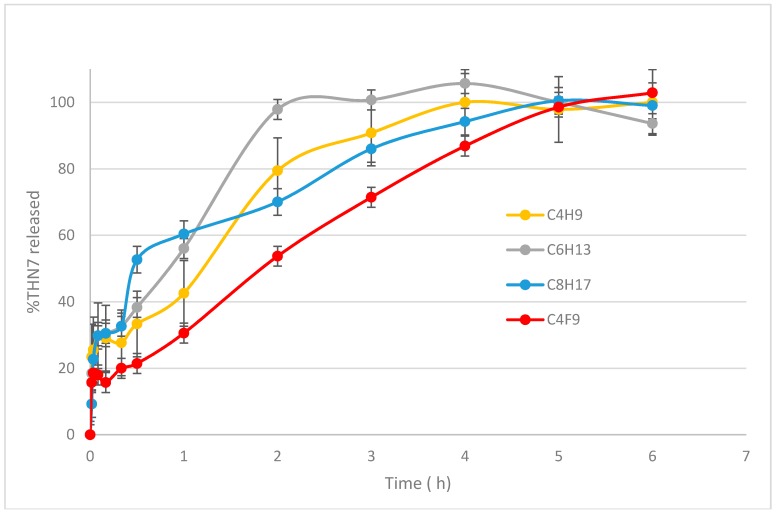
Release profiles of THN7 from loaded nanospheres. The data are the average of three experiments and the error bars indicate the three experiments.

The in vitro release experiments were performed in order to confirm what has previously been observed in similar systems: the benefit of nanoparticle encapsulation. Figure 3 displays the release profile of THN7 from loaded nanoparticles based on four α-CDs amphiphilic derivatives. 

In all cases, a fast release of up to 30% THN7 was observed within the first 30 min, followed by a much more controlled delivery of THN7, meaning that some of the THN7 molecules were probably coated on the surface of the nanospheres as expected before, and the remainder was solubilized inside the hydrophobic core of the nanoparticles. Complete drug release was reached in 1 to 5 h, the longer release being observed for the fluorinated derivative nanoparticles. This confirms the observations made by Krafft et al. [24] on the better resistance and low permeability of fluorinated colloidal systems.

### 2.4. Hemolytic Activity of α-CD Nanoparticles

**Figure 4:**
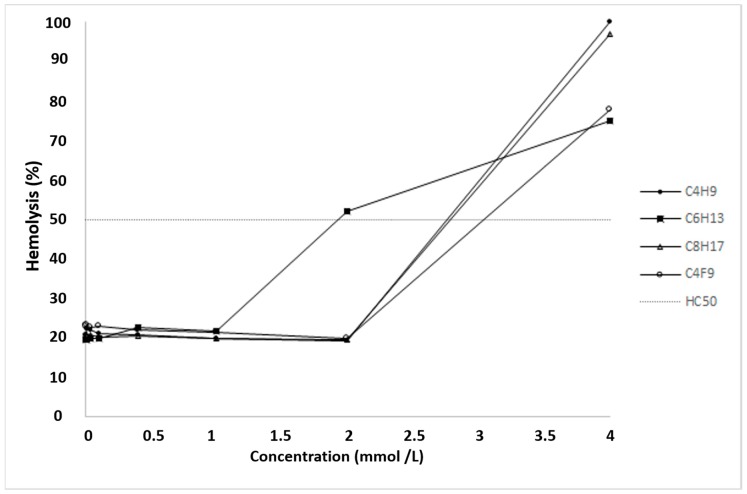
Hemolytic effect of different α-CD nanoparticles on human red blood cells (RBCs). Hemolysis was expressed as the percentage of the untreated control in the function of α-CD nanoparticle concentration. The positive control was purified water and the negative control was phosphate-buffered saline (PBS). Values presented are means ± SD. All data were obtained from three independent replicates, and in the same experiment, two to four parallel concentrations were measured. HC_50_ values were calculated as follows: C_6_H_13_ = 1.93 ± 0.11 mmol·L^−1^, C_4_H_9_ = 2.59 ± 0.8 mmol·L^−1^, C_8_H_17_ = 2.79 ± 0.75 mmol·L^−1^ and C_4_F_9_ = 3.04 ± 0.22 mmol·L^−1^.

C_4_F_9_ showed a relatively high hemolytic effect as its HC_50_ value was 3.04 ± 0.22 mmol·L^−1^ compared to the other derivatives. The ranking of the hemolytic effect in the case of other α-CD nanoparticles was classified as follows (smallest to largest): C_6_H_13_ > C_4_H_9_ > C_8_H_17_ (Figure 4).

### 2.5. Improved Cytotoxic Activity of THN7-Loaded α-Cyclodextrin Nanoparticles on A-427 Lung Cancer Cells

**Figure 5:**
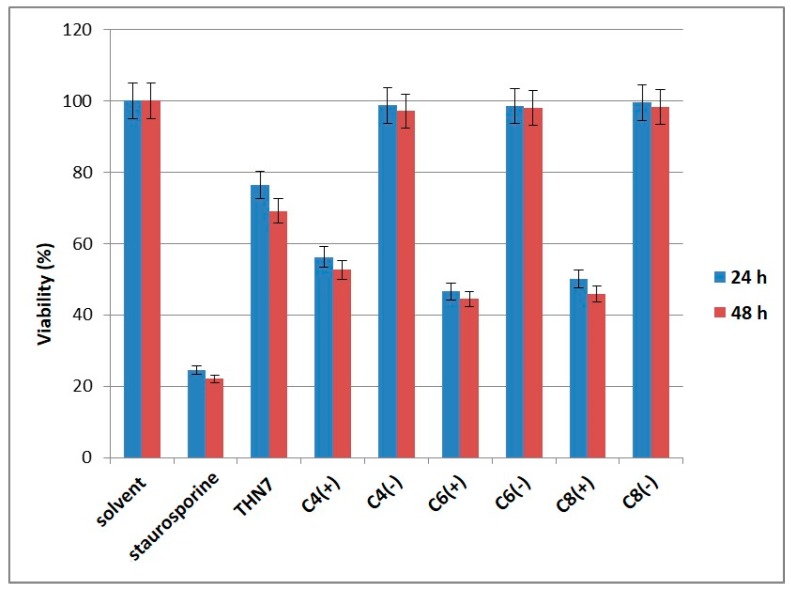
Cytotoxic activity of THN7-loaded α-cyclodextrin nanoparticles on A-427 lung cancer cells. C4: C_4_H_9_; C6: C_6_H_13_; C8: C_8_H_17_. (+) loaded cyclodextrins; (−) empty cyclodextrins. Viability of A427 cells were determined using an MTT assay. The viability of cells treated with 1% of the appropriate solvent was set 100%. Staurosporine served as a control for the correctly performance of the MTT assay using A427 cells. In all experiments THN7 was used at a final concentration of 6 μM.

To evaluate the cytotoxic effect of THN7-loaded α-cyclodextrin nanoparticles, we treated human lung cancer cells A427 with THN7 alone and each solution of THN7-loaded nanoparticles. In all experiments THN7 was used at a final concentration of 6 μM. A427 cells were selected as a model because they have been reported to overexpress CK2α, the target of kinase inhibitors from the class of indeno[1,2-*b*]indoles [25,26]. Moreover, other indenoindoles known to be potent CK2 inhibitors were described to have a significant antiproliferative effect on human A427 cells [8]. Cell viability of A427 cells was determined using an MTT assay, and the results are depicted in Figure 5.

The viability of A427 cells was decreased to 75% after treatment with 6 μM THN7 for 24 h. This effect is enhanced by an additional 20% in the case where cells were treated with α-cyclodextrin nanoparticles loaded with THN7. Empty nanoparticles had no effect on cell viability. An additional increase after 48 h incubation with or without nanoparticles was not observed.

After treatment with staurosporine at a concentration of 20 μM, cell viability was reduced to a residual 20%. This demonstrated that the MTT assay worked properly for the determination of cell viability of A427 cells. The cytotoxic effect of staurosporine on different cancer cell lines is well documented by many investigations [27,28]. 

## 3. Discussion

Amphiphilic α-cyclodextrin derivatives spontaneously formed nanoparticles in water and efficiently enclosed CK2 inhibitors. Unloaded cyclodextrin nanoparticles had no cytotoxic effect on A-427 lung cancer cells, while THN7-loaded α-cyclodextrin nanoparticles had a more pronounced cytotoxic effect after 24 and 48 h of exposure compared to THN7 alone. This phenomenon can be explained by several mechanisms. First of all, the solubilization of THN7 by amphiphilic cyclodextrins plays an important role in the improvement of its efficacy. The interaction of THN7 with cyclodextrin nanoparticles can prevent its aggregation in water, thus the CK2 inhibitor will penetrate freely through the cell membrane. On the other hand, it has also recently been revealed that both cyclodextrins [29,30] and poly-cationic amphiphilic cyclodextrin complexes [31] are able to enter the cells by endocytosis. Cellular internalization of loaded nanoparticles is a key step for their action and can highly increase the intracellular CK2 inhibitor concentration. To test and visualize the cellular internalization of amphiphilic α-cyclodextrin nanoparticles, the labeling of cyclodextrins with fluorophore is needed. Using fluorescent techniques, the interactions of nanoparticles with cells can be revealed. Our further aim is to functionalize the amphiphilic cyclodextrins by fluorophores and targeting moieties such as folates [32] or specific binding proteins. Targeting receptors or membrane proteins can improve the internalization efficiency, while by the fluorophores the intracellular distribution and fate of nanoparticles can be visualized and determined. A novel, multi-functional nanoscale drug delivery system, based on the nanoparticle formation of functionalized amphiphilic α-cyclodextrins, is under construction and will be tested on cancer cell lines. With a suitable targeting moiety, the in vivo efficacy, distribution, and toxicity of this carrier system can be characterized and used to improve the CK2 inhibitory effect on animal tumor models.

## 4. Materials and Methods 

### 4.1. Materials

All chemicals and phosphate buffer solution were purchased from Acros Organics or Sigma Aldrich and used without further purification. Other solvents were of chemical grade and were used as received. 4-methoxy-5-isopropyl-5,6,7,8-tetrahydroindeno[1,2-*b*]indole-9,10-dione (THN7) was synthetized according to the same procedure described by References [10,33]. The α-cyclodextrins were purchased from Roquette Frères (Lestrem, France) and their modification to amphiphilic CDs (Figure 2) was conducted according to the syntheses described previously by our group [34]. The structures and the purities of these amphiphilic derivatives were confirmed by spectral experiments according to techniques such as ^1^H and ^13^C NMR at 300 and 125 MHz, respectively, and MALDI mass spectroscopy. In order to simplify the name of each compound, shorter names have been assigned. For example, α-C_4_F_9_ refers to hexakis[6-deoxy-6-(3-perfluorobutylpropanethio)-2,3-di-*O*-methyl]-α-cyclodextrin.

### 4.2. Cell Culture

The nanoparticles were tested on the human lung cancer cell line A427, which was obtained from the German Collection of Microorganisms and Cell Culture (DSMZ, Braunschweig, Germany). A427 cells were grown at 37 °C in a humidified atmosphere of 5% CO_2_ in RPMI-1640 medium supplemented with 10% fetal calf serum (FCS).

### 4.3. Characterization of Inclusion Complexes Using the UV/Vis Spectroscopy

The continuous variation method was used to determine the stoichiometry of the fluorinated and hydrocarbonated amphiphilic cyclodextrin:THN7 complexes. Two solutions of equal concentration (4 × 10^−4^ M) of amphiphilic α-CDs and THN7 were prepared in ethanol. These solutions were mixed in different portions without variation of the final volume, and stirred for seven days. The absorbency (Aread) of each solution was measured at 460 nm. The absorbency change (∆A) was then calculated (∆A = Aread-AT). The Job plots showed a maximum at a specific molar ratio indicating the stoichiometry of the complexes (see supporting information). For the determination of the equilibrium constant (K), the double-reciprocal (Benesi/Hildebrand) plot was used as previously described [22].

### 4.4. Preparation of Nanoparticles

These results being the continuity of those published previously [20], we used the same highly loaded method: the THN7 loaded nanoparticles based on amphiphilic α-cyclodextrin derivatives were prepared by the nanoprecipitation technique, using 10 mL of a 4 × 10^−4^ M solution of preformed 1:1 THN7:CD complexes overloaded with an additional 20 mL of a 4 × 10^−4^ M THN7 solution in ethanol phase. This ethanolic solution (30 mL) was poured dropwise into deionized water (2 × 30 mL) and stirred at 400 rpm. Solvent and a part of water were evaporated under reduced pressure, and the total volume was adjusted to 20 mL with deionized water. The final concentration of THN7 in each nanoparticle stock solution was 600 μM. In addition, a DMSO stock solution of THN7 was prepared (also with a concentration of 600 μM).

### 4.5. Particle Size Measurements

The mean particle size (diameter, nm) and the polydispersity index (PDI) of nanospheres were measured by dynamic light scattering using a NanoZS instrument (Malvern Instruments, Malvern, UK) [22]. The measurements were carried out at 25 °C. Each value was the average of three measurements.

### 4.6. Determination of the Encapsulation Efficiency

For measuring the loading efficiency [12], after the formation of nanoparticle suspensions, unbound THN7 molecules in the nanoparticle dispersions were separated by centrifugation at 50,000 rpm for 1 h at +4 °C by an Optima™ MAX-E centrifuge (Beckman-Coulter), in order to settle down the loaded nanoparticles. The supernatant was removed. The precipitate was then dried overnight and the resulting powder containing the loaded nanoparticles was dissolved in ethanol to obtain a clear solution, the absorbance of which was analyzed using a spectrophotometer at a wavelength of 460 nm for the calculation of the encapsulated drug quantity. Experiments were conducted in triplicate. Loading capacity was expressed in terms of associated drug percentage:

Associated drug % = [determined drug quantity (μmol)]/[initial drug quantity (μmol)] × 100.

### 4.7. In Vitro-Controlled Release Profile

The suspensions of nanoparticles charged in THN7 (1 mL) were introduced into a dialysis tube (Sigma, Pur-A-Lyzer Mega 1000 Dialysis Kit, MWCO: 1 kDa). This tube was then placed in PBS (pH 7.4 with a volume of 10 mL), at room temperature, for a period of time. Aliquots of 1 mL of the buffered solution were taken from the media at specific time intervals and replaced by fresh PBS. The proportion of released and encapsulated THN7 molecules were measured by UV spectrometry at 460 nm [12]. Experiments were conducted three times.

### 4.8. Hemolysis Test

A hemolysis test was performed on fresh human blood, collected from healthy donors [35]. Erythrocytes were separated from citrated blood by centrifugation at 2500× *g* for 10 min, washed three times with PBS, and resuspended in the same solution. Aliquots of the cell suspension with the respective RBC number of 5 × 10^7^ were added to the PBS solution (pH 7.2) containing increasing concentrations of the samples investigated in the study. After mixing them gently, each solution was incubated at 37 °C for 10 min and then centrifuged at 5000× *g*. Finally, the absorbance of the hemoglobin released into the supernatant was measured at 540 nm with a FLUOstar OPTIMA Microplate Reader. The percentage of hemolysis was expressed as the ratio of hemoglobin in the supernatant of the sample solutions related to the hemoglobin concentration after the complete hemolysis of erythrocytes in water. The dose-response curve was determined, and the concentration inducing hemolysis in 50% of the erythrocyte population (HC_50_) was subsequently calculated [36]. 

### 4.9. Cell Viability Assay

For the determination of cell viability of A427 cells, an MTT assay was performed. A427 cells were seeded at a density of 10^4^ cells per well into 96-well plates in 100 μL of growth medium. After 24 h, the medium was replaced by fresh medium containing either a working solution containing THN7 alone (1 μL of THN7 stock solution added to 99 μL medium) or a working solution of THN7-loaded nanoparticles (1 μL of stock solution of THN7-loaded nanoparticles added to 99 μL medium). Then, in each well the final concentration of THN7 (alone or in nanoparticle solution) was 6 μM. Blank experiments were also conducted with empty cyclodextrins. Medium containing 1% of the solvent served as a control. The concentration of staurosporin used in the experiment was 0.5 µM. After 24 h and 48 h, respectively, 10 μL of the MTT solution (5 mg/mL in PBS) were added to each well and the plate was incubated for 2 h at 37 °C. The MTT assay is based on the ability of the mitochondrial succinate-tetrazolium reductase system to convert 3-(4,5-dimethylthiazol-2-yl)-2,5-diphenyltetrazolium bromide (MTT) to the purple-colored formazan. The produced formazan was dissolved by the addition of 100 μL of the MTT stop solution (0.04 M HCl in isopropanol) and the plates were incubated for one additional hour at 37 °C before recording the absorbance at 570 nm.

## 5. Conclusions

The development of advanced drug delivery systems dedicated to kinase inhibitors is important to investigate for enhancing the uptake of anticancer drugs into tumors. In the present study, four α-CDs were used to encapsulate an indenoindole-type CK2 inhibitor, THN7. All experiments indicated that the nanoencapsulation of THN7 in α-CDs was successful. In parallel, after testing the hemolytic activity of the four selected α-CDs, only three THN7-loaded cyclodextrin nanoparticles (α-C_6_H_13_, α-C_8_H_17_, and α-C_4_H_9_) were tested against A-427 cells. It is important to note that all THN7-loaded nanoparticles were more cytotoxic against A-427 cells than THN7 alone. 

Further studies should be investigated to optimize the drug-loading capacity of our amphiphilic α-cyclodextrin-based nanoparticles. In the present study, we only obtained an encapsulation rate of 35% with the α-C_6_H_13_ amphiphilic derivative. The structural modulation of both amphiphilic α-cyclodextrins and indeno[1,2-*b*]indoles could be rapidly achieved, giving new insight into amphiphilic α-cyclodextrin derivatives for targeted drug delivery to tumors. As suggested in the perspectives, we will examine how amphiphilic α-cyclodextrins could assist the bioactive molecule (in this case, a CK2 inhibitor) to enhance in cellulo activity against cancer cells (e.g., A-427, MCF-7). This is a crucial point to study before undertaking an in vivo development project. 

## Figures and Tables

**Table 1:** Stoichiometry and stability constants (K_11_) of complexes in ethanol.

α-Cyclodextrin	Stoichiometry	K_11_ (mol·L^−1^)
C_4_H_9_	1:1	057
C_6_H_13_	1:1	298
C_8_H_17_	1:1	417
C_4_F_9_	1:1	828

**Table 2:** Mean diameter (nm) and polydispersity index (PdI) of nanospheres based on different amphiphilic α-cyclodextrins and % of associated THN7.

α-Cyclodextrin	Nanosphere Size (nm)	PdI	Associated THN7 (%)
C_4_H_9_	82.2 ± 0.2	0.07 ± 0.01	24 ± 3
C_6_H_13_	131.9 ± 0.4	0.07 ± 0.03	35 ± 2
C_8_H_17_	65.8 ± 3.0	0.18± 0.02	14 ± 5
C_4_F_9_	104.3 ± 1.0	0.09 ± 0.02	19 ± 3

## Supplementary Materials

The following are available online at http://www.mdpi.com/1424-8247/11/1/10/s1. Figure S1: Job plot for the complexation of THN7 with α-C_4_H_9_ amphiphilic derivative, Figure S2: Job plot for the complexation of THN7 with α-C_6_H_13_ amphiphilic derivative, Figure S3: Job plot for the complexation of THN7 with α-C_8_H_17_ amphiphilic derivative, Figure S4: Job plot for the complexation of THN7 with α-C_4_F_9_ amphiphilic derivative, Figure S5: Benesi-Hildebrand plot for amphiphilic α-cyclodextrins. THN7 at constant concentration in the presence of increasing concentrations of amphiphilic α-CD derivatives at 256 nm, Figure S6: Dynamic light scattering experiments spectra and mean diameter of THN7-loaded C_4_H_9_ amphiphilic CDs, Figure S7: Dynamic light scattering experiments spectra and mean diameter of THN7-loaded C_6_H_13_ amphiphilic CDs, Figure S8: Dynamic light scattering experiments spectra and mean diameter of THN7-loaded C_8_H_17_ amphiphilic CDs, Figure S9: Dynamic light scattering experiments spectra and mean diameter of THN7-loaded C_4_F_9_ amphiphilic CDs.
