# Supplementary material for: Self-Assembled Supramolecular Nanoparticles Improve the Cytotoxic Efficacy of CK2 Inhibitor THN7
Source: Pharmaceuticals (Basel). 2018 Jan 26;11(1):10. doi: 10.3390/ph11010010 (PMC5874706; doi:10.3390/ph11010010)
Supplement: Supplementary file 1 [file pharmaceuticals-11-00010-s001.pdf]

Appendix A. Supplementary Materials

Self-assembled supramolecular nanoparticles improve the cytotoxic efficacy of CK2 inhibitor THN7

Abdelhamid Nacereddine [1,2], Andre Bollacke [2], Eszter Róka [3,4], Christelle Marminon [1], Zouhair Bouaziz [1], Ferenc Fenyvesi [3], Ildikó Katalin Bácskay [3], Joachim Jose [2], Florent Perret [4,*] and Marc Le Borgne [2,*]

[1] Faculté de Pharmacie—ISPB, EA 4446 Bioactive Molecules and Medicinal Chemistry, SFR Santé Lyon-Est CNRS UMS3453—INSERM US7, Université Claude Bernard Lyon 1, Université de Lyon, 8 Avenue Rockefeller, F-69373 Lyon CEDEX 8, France; a.nacereddine@gmail.com (A.N.); christelle.marminon-davoust@univ-lyon1.fr (C.M.); zouhair.bouaziz@univ-lyon1.fr (Z.B.)

[2] Institute of Pharmaceutical and Medicinal Chemistry, PharmaCampus, Westfälische Wilhelms-Universität Münster, Corrensstr. 48, 48149 Münster, Germany; andre.bollacke@uni-muenster.de (A.B.); joachim.jose@uni-muenster.de (J.J.)

[3] Department of Pharmaceutical Technology, Faculty of Pharmacy, University of Debrecen, Nagyerdei körút 98, H-4032 Debrecen, Hungary; eszter.roka@gmail.com (E.R.); fenyvesi.ferenc@pharm.unideb.hu (F.F.); bacskay.ildiko@pharm.unideb.hu (I.K.B.)

[4] CSAp, Institut de Chimie et Biochimie Moléculaires et Supramoléculaires, Bâtiment Raulin, Université de Lyon, Université Claude Bernard Lyon 1, 43 Bd du 11 novembre 1918, 69622 Villeurbanne CEDEX, France

* Correspondence: florent.perret@univ-lyon1.fr (F.P.); marc.le-borgne@univ-lyon1.fr (M.L.B.); Tel.: +33-4-72-43-28-25 (F.P.); +33-4-78-77-75-42 (M.L.B.)

CAPTIONS:

Figure S1. Job plot for the complexation of THN7 with α-C_4H_9 amphiphilic derivative.

Figure S2. Job plot for the complexation of THN7 with α-C_6H_{13} amphiphilic derivative.

Figure S3. Job plot for the complexation of THN7 with α-C_8H_{17} amphiphilic derivative.

Figure S4. Job plot for the complexation of THN7 with α-C_4F_9 amphiphilic derivative.

Figure S5. Benesi-Hildebrand plot for amphiphilic α-cyclodextrins. THN7 at constant concentration in the presence of increasing concentrations of amphiphilic α-CD derivatives at 256 nm.

Figure S6. Dynamic light scattering experiments spectra and mean diameter of THN7 loaded C_4H_9 amphiphilic CDs.

Figure S7. Dynamic light scattering experiments spectra and mean diameter of THN7 loaded C_6H_{13} amphiphilic CDs.

Figure S8. Dynamic light scattering experiments spectra and mean diameter of THN7 loaded C_8H_{17} amphiphilic CDs.

Figure S9. Dynamic light scattering experiments spectra and mean diameter of THN7 loaded C_4F_9 amphiphilic CDs.

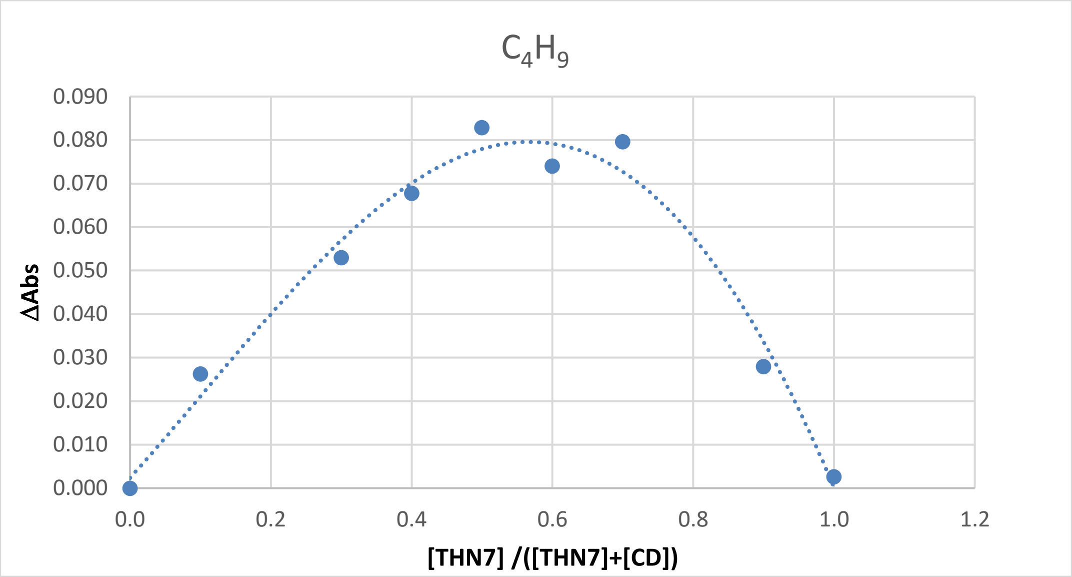

Figure S1. Job plot for the complexation of THN7 with α-C_4H_9 amphiphilic derivative.

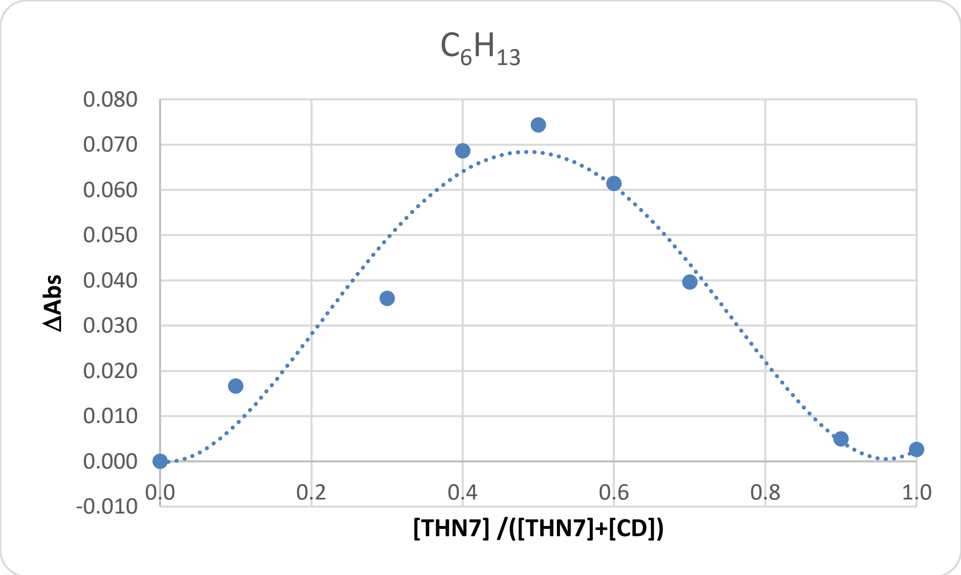

Figure S2. Job plot for the complexation of THN7 with α-C_6H_{13} amphiphilic derivative.

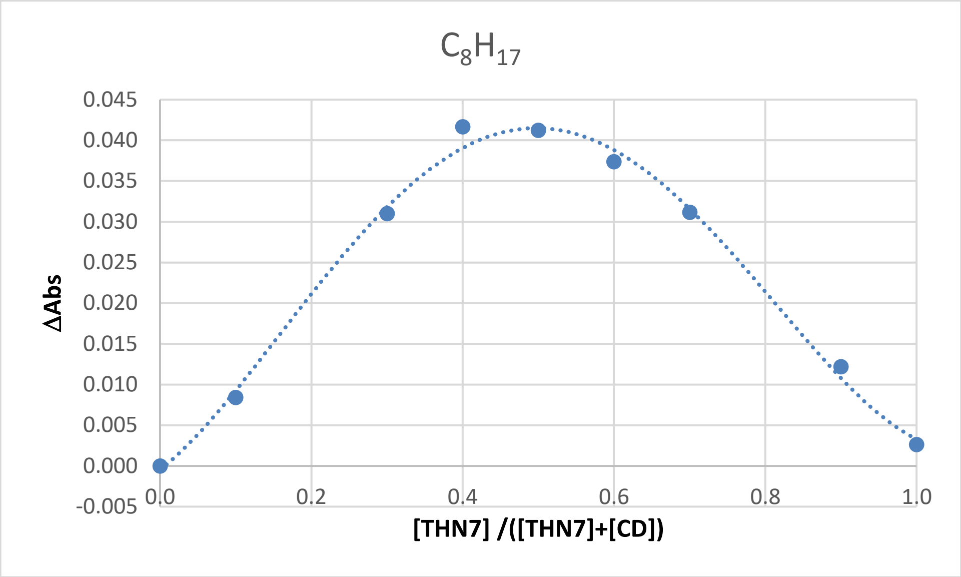

Figure S3. Job plot for the complexation of THN7 with α-C_8H_{17} amphiphilic derivative.

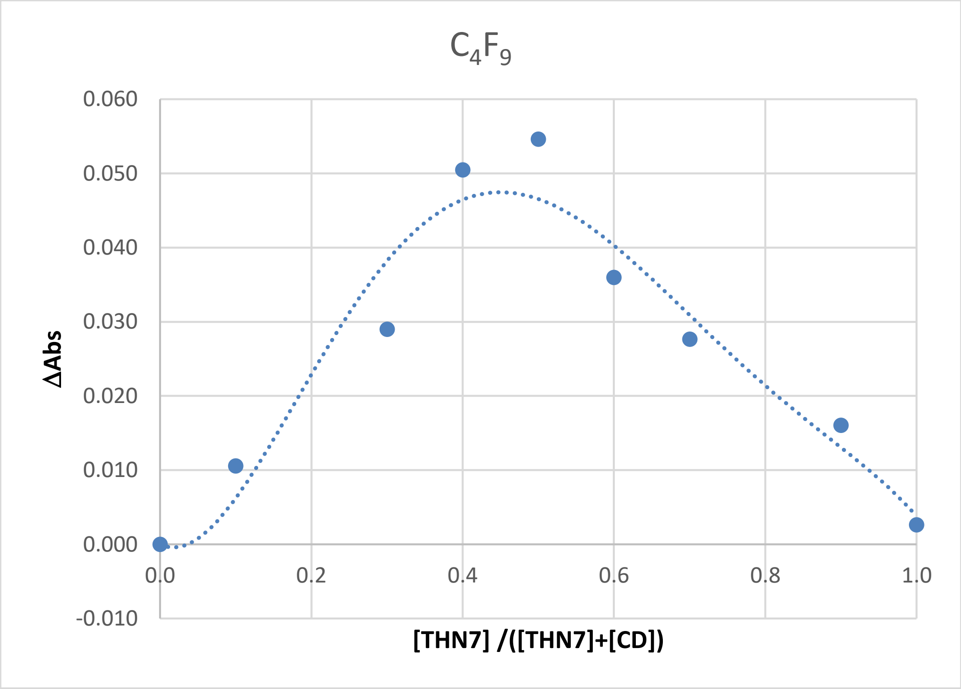

Figure S4. Job plot for the complexation of THN7 with α-C_4F_9 amphiphilic derivative.

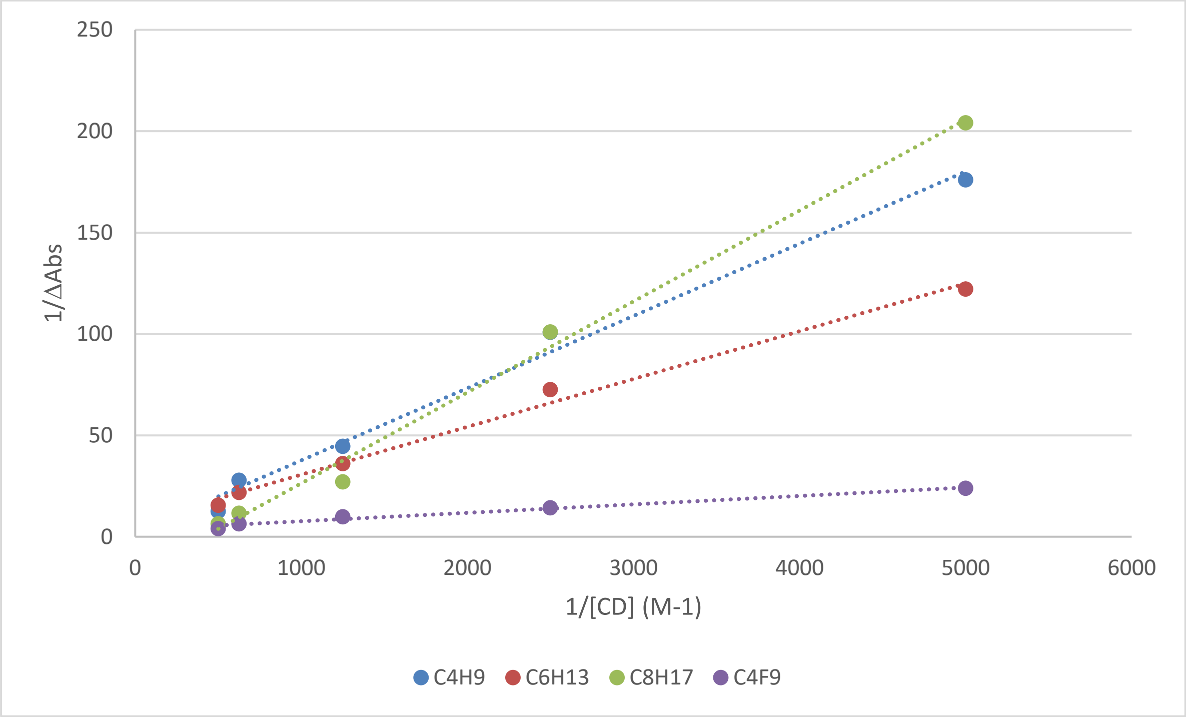

Figure S5. Benesi-Hildebrand plot for amphiphilic α-cyclodextrins. THN7 at constant concentration in the presence of increasing concentrations of amphiphilic α-CD derivatives at 460 nm.

Results

			Size (d.n...	% Intensity:	St Dev (d.n...
Z-Average (d.nm):	82,03	Peak 1:	85,62	100,0	20,11
PdI:	0,075	Peak 2:	0,000	0,0	0,000
Intercept:	0,966	Peak 3:	0,000	0,0	0,000
Result quality	Good				

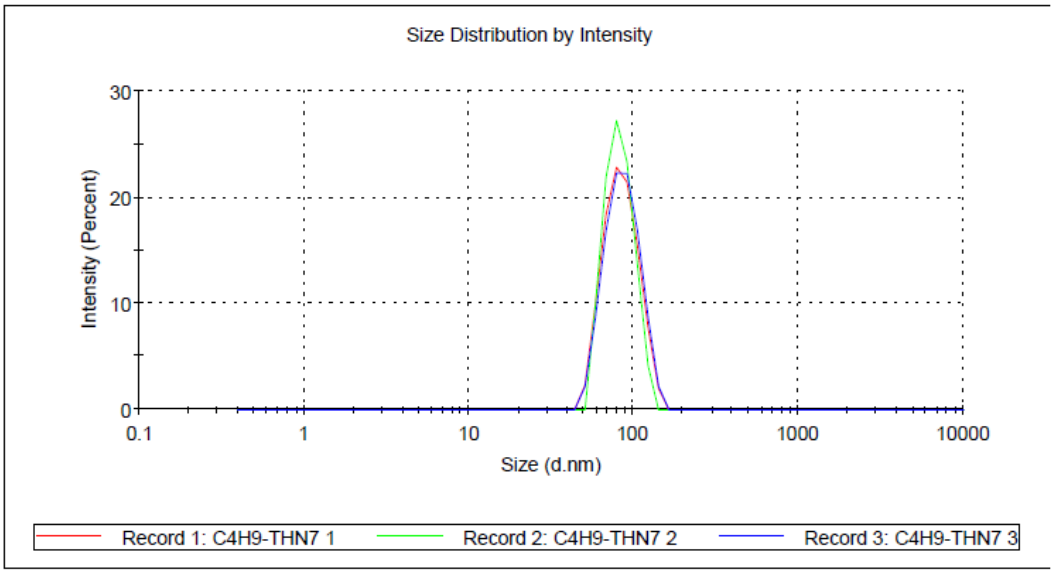

Figure S6. Dynamic light scattering experiments spectra and mean diameter of THN7 loaded C_4H_9 amphiphilic CDs.

Results

			Size (d.n...	% Intensity:	St Dev (d.n...
Z-Average (d.nm):	132,3	Peak 1:	143,6	100,0	41,65
PdI:	0,084	Peak 2:	0,000	0,0	0,000
Intercept:	0,962	Peak 3:	0,000	0,0	0,000
Result quality	Good				

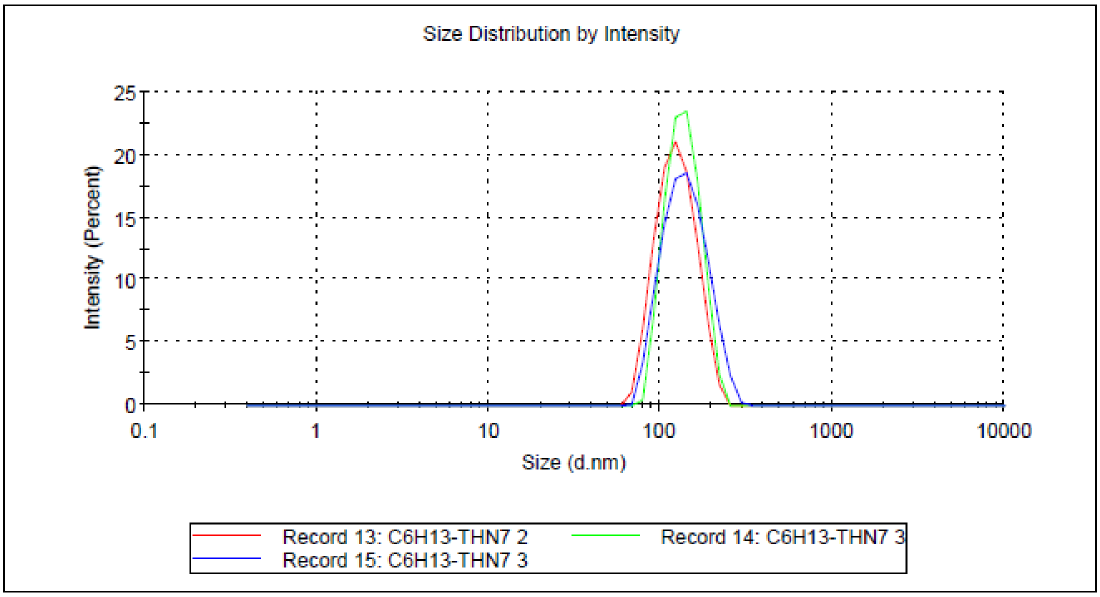

Figure S7. Dynamic light scattering experiments spectra and mean diameter of THN7 loaded C_6H_{13} amphiphilic CDs.

Results

			Size (d.n...	% Intensity:	St Dev (d.n...
Z-Average (d.nm):	68,81	Peak 1:	60,11	100,0	13,23
PdI:	0,198	Peak 2:	0,000	0,0	0,000
Intercept:	0,938	Peak 3:	0,000	0,0	0,000
Result quality	Refer to quality report				

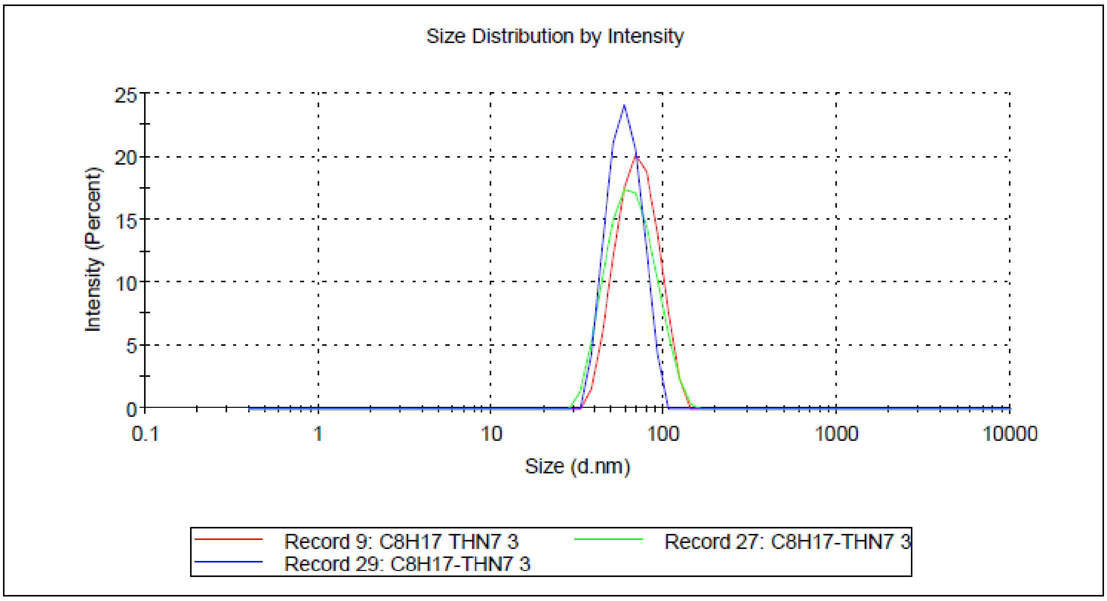

Figure S8. Dynamic light scattering experiments spectra and mean diameter of THN7 loaded C_8H_{17} amphiphilic CDs.

Results

			Size (d.n...	% Intensity:	St Dev (d.n...
Z-Average (d.nm):	103,3	Peak 1:	114,5	100,0	37,77
PdI:	0,103	Peak 2:	0,000	0,0	0,000
Intercept:	0,963	Peak 3:	0,000	0,0	0,000
Result quality	Good				

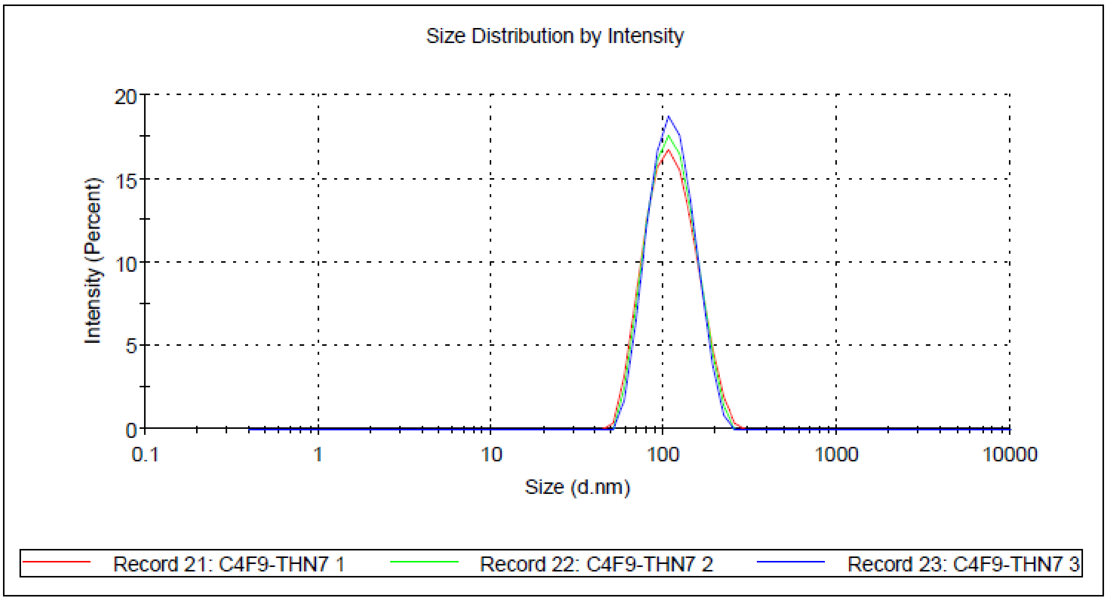

Figure S9. Dynamic light scattering experiments spectra and mean diameter of THN7 loaded C_4F_9 amphiphilic CDs.
